# Editorial: The pharmacological modulation of angiogenesis

**DOI:** 10.3389/fphar.2024.1474918

**Published:** 2024-08-27

**Authors:** Emanuela Pessolano, Raffaella Belvedere, Simona Federica Spampinato

**Affiliations:** ^1^ Department of Pharmaceutical Sciences, Università del Piemonte Orientale, Novara, Italy; ^2^ Department of Pharmacy, University of Salerno, Fisciano, Salerno, Italy; ^3^ Department of Scienza e Tecnologia del Farmaco, University of Turin, Turin, Italy

**Keywords:** angiogenesis, tumor vasculature, diabetic retinopathy, corneal neovascularisation, pharmacological modulation

This editorial aims to provide an overview of the complex process of angiogenesis and its implications for human health. Angiogenesis is a complex process with far-reaching implications for human health. Its dysregulation can contribute to a myriad of diseases, including cancer, diabetic retinopathy, and impaired wound repair (Pessolano et al., 2018; Pessolano et al., 2021; Belvedere et al., 2022). Huang et al. demonstrated melatonin’s efficacy in promoting angiogenesis, suggesting a promising avenue for reconstructive surgery. Indeed, in his work, Ribatti’s points the attention on tumor vasculature, revealing critical differences between healthy and cancerous vessels, offering targets for anti-cancer therapies. Finally, the importance of the pharmacological modulation of angiogenesis in pathological conditions is explored. Ocular neovascularization represents an important Research Topic in the loss of vision. The anti-angiogenic and antioxidant effects of axitinib are analysed and suggested as potential treatment for diabetic retinopathy by Lazzara et al.

Deng et al. instead focuses on the ability of LSD-1 in controlling corneal neovascularization, identifying a potential therapeutic target. These studies collectively underscore the multifaceted nature of angiogenesis and its potential as a therapeutic target across various diseases.
